# Association between single nucleotide polymorphisms in the *TSPYL6* gene and breast cancer susceptibility in the Han Chinese population

**DOI:** 10.18632/oncotarget.10754

**Published:** 2016-07-21

**Authors:** Ming Liu, Bin Li, Wen Guo, Xiyang Zhang, Zhengshuai Chen, Jingjie Li, Mengdan Yan, Chao Chen, Tianbo Jin

**Affiliations:** ^1^ School of Life Sciences, Northwest University, Xi'an 710069, China; ^2^ National Engineering Research Center for Miniaturized Detection Systems, Xi'an 710069, China; ^3^ Department of Obstetrics and Gynecology, Second Affiliated Hospital, Xi'an Jiaotong University, Xi'an 710004, China; ^4^ Inner Mongolia Medical University, Inner Mongolia, Hohhot 010050, China

**Keywords:** association study, breast cancer, TSPYL6, single nucleotide polymorphism

## Abstract

We investigated the associations between single nucleotide polymorphisms (SNPs) in the *testis-specific Y-encoded-like protein 6* (*TSPYL6*) *gene* and breast cancer (BC) susceptibility in the Han Chinese population. A total of 183 BC patients and 195 healthy women were included in the study. Six SNPs in *TSPYL6* were genotyped and the association with BC risk analyzed. Odds ratios (ORs) and 95% confidence intervals (95% CIs) were calculated using unconditional logistic regression analysis. Multivariate logistic regression analysis was used to identify SNPs that correlated with BC susceptibility. Rs11896604 was associated with a decreased risk of BC based on dominant and genotype models. Rs843706 was associated with an increased risk of BC based on a recessive model. Rs11125529 was associated with decreased BC susceptibility based on a genotype model. Finally, rs843711 inversely correlated with clinical stage III/IV BC. Our findings reveal a significant association between SNPs in the *TSPYL6* gene and BC risk in a Han Chinese population.

## INTRODUCTION

Breast cancer (BC) is the most common type of cancer and the leading cause of cancer deaths among women worldwide (particularly in less developed regions including East Asian countries, which accounted for 324,000 deaths or 14.3% of the total) [1]. According to GLOBOCAN 2012, 187,213 individuals were diagnosed with BC in China in 2012, and 47,984 of these individuals died of the disease [2]. BC is a multifactorial disease that has been associated with various factors including age, gender, ethnicity, family history, personal history, lifestyle, as well as both hormonal and non-hormonal risk factors [3]. Hereditary BC clusters in families and is typically diagnosed at an earlier age [4]. Studies of twins have indicated that the risk of BC is higher for a monozygotic twin of a co-twin, suggesting that genetic factors play an important role in BC development [5]. Single nucleotide polymorphisms (SNPs) also play an important role in the genetic susceptibility to BC. Many genes have been associated with a moderate or high lifetime risk of BC including *BRCA1*, *BRCA2*, *PALB2, ATM*, and *CHEK2*. In addition, common variants at more than 70 loci have been identified through GWAS and large-scale replication studies [6–9].

The testis-specific Y-encoded-like protein 6 (*TSPYL6*) gene, located on human chromosome 2p16.2, is a member of the *TSPY/TSPYL/SET/NAP-1* (TTSN) superfamily that includes *TSPYL1, TSPYL2, TSPYL3, TSPYL4,* and *TSPYL5* [10]. Upregulation of *TSPYL6* has been observed in both benign and malignant cells. The *TSPYL6* protein has been associated with chromatin and nucleosome assembly [11]. However, the specific functions of *TSPYL6* are not yet clear. Norling et al. [12] sequenced the *TSPYL6* gene in an entire Sweden patient cohort, but no inactivating mutations were identified. Additionally, no studies have investigated correlations between the *TSPYL6* gene and BC susceptibility. In this case-control study, we genotyped six SNPs in *TSPYL6*: rs843645, rs11125529, rs12615793, rs843711, rs11896604, and rs843706 and performed a comprehensive association analysis to identify SNPs associated with BC risk in Han Chinese women.

## RESULTS

### Participant characteristics

A total of 183 patients with BC and 195 healthy individuals were enrolled in the study. The participant characteristics are shown in Table 1. No significant differences in age, body mass index (BMI), or the menopause age were observed between patients in the case and control groups (*p* > 0.05). The mean age of the participants was 45.35 years in the control group and 46.40 years in the case group. The mean BMI was 22.53 in the control group and 23.08 in the case group.

**Table 1:** Basic characteristics of the control individuals and patients with breast cancer

Characteristic		Cases (*N* = 183)	Controls (*N* = 195)	*P* -value
Mean age ± SD		46.40 ± 9.383 (*N*= 183)	45.35 ± 6.899 (*N*= 195)	0.218^a^
Mean BMI ± SD		23.08 ± 3.00 (*N*= 183)	22.53 ± 2.55 (*N* = 195)	0.056^a^
Menopause	Premenopausal	115 (62.8%)	119 (61.0%)	0.716^b^
	Postmenopausal	68 (37.2%)	76 (39.0%)	
Age of Menarche	≤ 12	25 (13.7%)		
	> 12	158 (86.3%)		
Breastfeeding Duration	≤ 6	12 (6.5%)		
	> 6	158 (93.5%)		
Clinical Stages	I/II	135 (73.8%)		
	III/IV	48 (26.2%)		
Estrogen Receptor	negative	60 (32.8%)		
	positive	123 (67.2%)		
Family Tumor History	no	156 (85.2%)		
	yes	27 (14.8%)		
Incipientw or Recurrence	Incipient	109 (59.9%)		
	Recurrence	73 (40.1%)		
Lymph node metastasis	no	105 (58.3%)		
	yes	75 (41.7%)		
Menopause	no	115 (62.8%)		
	yes	68 (37.2%)		
Primiparous Age	< 30	170 (96.6%)		
	≥ 30	6 (3.4%)		
Procreative Times	< 1	142 (81.1%)		
	≥ 1	33 (18.9%)		
Progestrone Receptor	negative	75 (41.0%)		
	positive	108 (59.0%)		
Tumor Location	left	84 (45.9%)		
	right	97 (53.0%)		
	both	2 (1.1%)		
Tumor Size (cm)	≤ 3	94 (51.4%)		
	> 3	89 (48.6%)		
Tumor Type	carcinoma	165 (90.2%)		
	others	18 (9.8%)		
Whether fertility	no	7 (3.8%)		
	yes	176 (96.2%)		

SD: Standard deviation. BMI: Body mass index (weight [kg]/height[m]^2^).

a *P* value was calculated by Welch's *t* test.

b *P* value was calculated by Pearson's χ^2^ test.

### Association between *TSPYL6* polymorphisms and BC risk

Detailed SNP data and the associations between various SNPs and BC risk are shown in Table 2. Our data indicated that all 6 SNPs investigated were in Hardy-Weinberg equilibrium in the control subjects (*p* > 0.05). No associations were observed between the alleles and BC risk in an allele model. We also performed a Bonferroni correction and determined that none of the SNPs showed statistical significant associations with BC risk.

**Table 2:** Basic information of candidate SNPs in this study

SNPs	Position	Band	Alleles^A^/^B^	MAF-control	MAF-case	HWE-p	OR	95% CI	*p*-x^2^
rs843645	54474664	2p16.2	G/T	0.297	0.279	0.4968	0.913	0.666–1.251	0.57
rs11125529	54475866	2p16.2	A/C	0.195	0.150	0.1695	0.731	0.499–1.069	0.105
rs12615793	54475914	2p16.2	A/G	0.201	0.161	0.1166	0.764	0.525–1.109	0.156
rs843711	54479117	2p16.2	C/T	0.487	0.544	0.1531	1.254	0.942–1.669	0.12
rs11896604	54479199	2p16.2	G/C	0.221	0.167	0.0929	0.707	0.491–1.018	0.062
rs843706	54480369	2p16.2	C/A	0.482	0.544	0.06103	1.281	0.962–1.705	0.090

SNPs: Single nucleotide polymorphisms; MAF: Minor allele frequency; HWE: Hardy-Weinberg equilibrium; OR: Odds ratio; CI: Confidence interval.

A Minor alleles.

B Major alleles.

We further assessed the association between each SNP and BC risk in an unconditional logistic regression analysis, which was performed using four models: additive, dominant, recessive, and genotype model (Tables 3 and 4). Rs11896604 was associated with a decreased risk of BC in a dominant model (odds ratio [OR] = 0.623, 95% confidence interval [95% CI] = 0.405–0.958, *p* = 0.031). Rs843706 was associated with an increased risk of BC under the recessive model (OR = 1.709, 95% CI = 1.055–2.770, *p* = 0.030) (Table 3). Rs11125529 was associated with a decreased risk of BC under the genotype model (OR = 0.612, 95% CI = 0.391–0.959, *p* = 0.032) (Table 4). Rs11896604 was associated with a decreased risk of BC in a genotype model (OR = 0.574, 95% CI = 0.370–0.891, *p* = 0.013). No statistical associations were detected under the other models. In addition, no positive results were observed after Bonferroni correction.

**Table 3:** Single loci association with breast cancer risk (adjusted by age, BMI and menopause)

SNP	Model	Genotype	Cases	Controls	OR (95% CI)	*P*
rs843645	Dominant model	T/T	99	94	1	0.246
		G/G-G/T	84	101	0.785 (0.521–1.182)	
	Recessive model	T/T-T/G	165	180	1	0.535
		G/G	18	15	1.259 (0.608–2.606)	
	Additive model	-	-	-	0.904 (0.658–1.241)	0.532
rs11125529	Dominant model	C/C	133	123	1	0.057
		A/A-A/C	50	72	0.652 (0.419–1.013)	
	Recessive model	C/C-C/A	178	191	1	0.618
		A/A	5	4	1.413 (0.364–5.488)	
	Additive model	-	-	-	0.730 (0.491–1.085)	0.120
rs12615793	Dominant model	G/G	129	120	1	0.085
		A/A-A/G	54	74	0.682 (0.441–1.054)	
	Recessive model	G/G-G/A	178	190	1	0.625
		A/A	5	4	1.402 (0.361–5.445)	
	Additive model	-	-	-	0.755 (0.510–1.117)	0.159
rs843711	Dominant model	T/T	39	46	1	0.532
		C/C-C/T	144	149	1.169 (0.405–0.958)	
	Recessive model	T/T-T/C	128	154	1	0.066
		C/C	55	41	1.563 (0.972–2.515)	
	Additive model	-	-	-	1.261 (0.937–1.700)	0.126
rs11896604	Dominant model	C/C	128	114	1	**0.031**
		G/G-G/C	55	81	0.623 (0.405–0.958)	
	Recessive model	C/C-C/G	177	190	1	0.644
		G/G	6	5	1.336 (0.391–4.564)	
	Additive model	-	-	-	0.709 (0.484–1.039)	0.078
rs843706	Dominant model	A/A	39	45	1	0.603
		C/C-C/A	144	149	1.140 (0.696–1.866)	
	Recessive model	A/A-A/C	128	156	1	**0.030**
		C/C	55	38	1.709 (1.055–2.770)	
	Additive model	-	-	-	1.294 (0.958–1.750)	0.093

SNPs: Single nucleotide polymorphisms; OR: Odds ratio. CI: Confidence interval.

*P* value was calculated by Wald test. *p < 0.05 indicates statistical significant.

**Table 4:** The association between the single-nucleotide polymorphisms and BC risk in Genotype model (adjusted by age, BMI and menopause)

Genotype	Cases	Controls	OR (95% CI)	*P*
rs843645				
TT	99	94	1.00 [Ref]	
GT	66	86	0.729 (0.475–1.117)	0.147
GG	18	15	1.139 (0.543–2.391)	0.730
rs11125529				
CC	133	123	1.00 [Ref]	
AC	45	68	0.612 (0.391–0.959)	**0.032**
AA	5	4	1.156 (0.304–4.404)	0.832
rs12615793				
GG	129	120	1.00 [Ref]	
AG	49	70	0.651 (0.419–1.013)	0.057
AA	5	4	1.163 (0.305–4.432)	0.825
rs843711				
TT	39	46	1.00 [Ref]	
CT	89	108	0.972 (0.583–1.620)	0.913
CC	55	41	1.582 (0.879–2.848)	0.126
rs11896604				
CC	128	114	1.00 [Ref]	
GC	49	76	0.574 (0.37–0.891)	0.013
GG	6	5	1.069 (0.318–3.596)	0.915
rs843706				
AA	39	45	1.00 [Ref]	
CA	89	111	0.925 (0.555–1.543)	0.766
CC	55	38	1.670 (0.921–3.030)	0.092

OR: odd ratio; CI: confidence interval;

*p* value was calculated by Wald test. *p < 0.05 indicates statistical significance.

**Figure 1:**
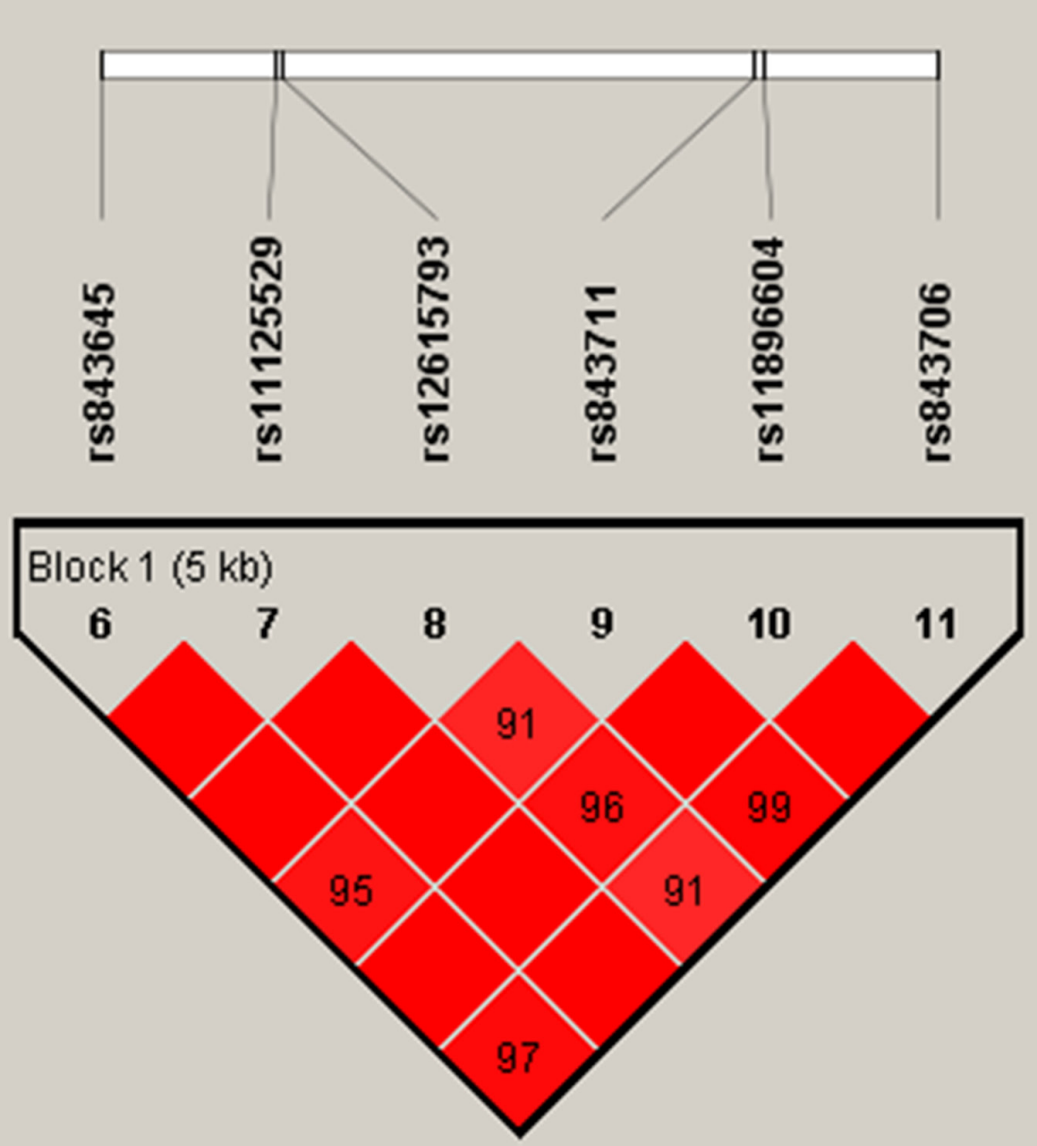
Haplotype block map for all the SNPs of the *TSPYL6* gene

In order to assess the associations between SNP haplotypes and BC risk, a Wald test was performed using an unconditional multivariate regression analysis. However, no positive results were observed (Table 5, Figure 1).

**Table 5:** Haplotype frequency and their association with BC risk in case and control subjects (adjusted by age, BMI and menopause)

SNPs	Haplotype	Freq %		*P1*	OR	95% CI		*P2*
		case	control					
rs843645|rs11125529|rs12615793|rs8437 11|rs11896604|rs843706	TAATGA	0.150	0.192	0.126	0.745	0.501	1.108	0.146
	TCGTGA	0.016	0.023	0.510	0.741	0.256	2.149	0.581
	GCGTCA	0.276	0.292	0.619	0.912	0.662	1.257	0.574
	TCGCCC	0.530	0.474	0.126	1.266	0.939	1.707	0.122

*P-value < 0.05 indicates statistical significance.

*P1*- values were calculated from two-sided Chi-squared test.

*P2* -values were calculated by unconditional logistic regression.

The reference standard for each haplotype is the other haplotype.

### Association between *TSPYL6* polymorphisms and BC patient clinicopathological features

We next analyzed the association between *TSPYL6* polymorphisms and BC patient clinicopathological features, which included age, age of menarche, BMI, breastfeeding duration, clinical stage, estrogen receptor status, family history of cancer, procreative time, progesterone receptor status, tumor location, tumor size (cm), tumor type, incipient recurrence, presence of lymph node metastasis, age of menopause, and prim parous age. Positive results are shown in (Table 6A, 6B). For rs11125529, we found that more recurrent BC patients had the AA + CA genotype than the CC genotype (OR = 2.321, 95% CI = 1.192–4.521, *p* = 0.012) (Table 6A). For rs843711, the CT + CC genotype was observed less frequently in patients with clinical stage III/IV disease (OR = 0.411, 95% CI = 0.194–0.869, *p* = 0.018) and in patients with recurrent BC (OR = 0.458, 95% CI = 0.222–0.944, *p* = 0.032) than the TT genotype (Table 6A). Our results suggested that the frequency of recurrent BC patients with the CC genotype of rs11896604 was higher than the frequency of patients with the GG + CG genotype (OR = 2.471, 95% CI = 1.290–4.734, *p* = 0.006) (Table 6B). Finally, the CA + CC genotype of rs843706 was more frequently observed in patients with clinical stage III/IV disease (OR = 0.411, 95% CI = 0.194–0.869, *p* = 0.018) and in patients with recurrent BC (OR = 0.458, 95% CI = 0.222–0.944, *p* = 0.032) than the AA genotype (Table 6B). No statistical associations were detected between the other loci and the clinical parameters that were investigated.

**Table 6A:** The Associations between *TSPYL6* polymorphisms and clinical characteristics of breast cancer patients

Variables	rs11125529					rs843711				
	AA + CA	CC	OR^a^	95% CI	*P*^b^	CT+CC	TT	OR^a^	95% CI	*P*^b^
Age	50	133				144	39			
≤ 40	14	43	1	(reference)		41	16	1	(reference)	
> 40	36	90	1.229	(0.600–2.515)	0.573	103	23	1.748	(0.839–3.639)	0.133
Age of Menarche	50	133				144	39			
≤ 12	5	20	1	(reference)		22	3	1	(reference)	
> 12	45	113	0.628	(0.222–1.775)	0.377	122	36	2.164	(0.612–7.646)	0.221
BMI	50	133				144	39			
≤ 24	34	87	1	(reference)		98	23	1	(reference)	
> 24	16	46	0.89	(0.445–1.780)	0.742	46	16	0.675	(0.326–1.397)	0.288
Breastfeeding Duration	46	124				133	37			
≤ 6	3	9	1	(reference)		10	2	1	(reference)	
> 6	43	115	0.891	(0.230–3.448)	0.868	123	35	1.423	(0.298–6.797)	0.657
Clinical Stages	50	133				144	39			
I/II	38	97	1	(reference)		112	23	1	(reference)	
III/IV	12	36	0.851	(0.401–1.807)	0.674	32	16	0.411	(0.194–0.869)	0.018*
Estrogen Receptor	50	133				144	39			
negative	17	43	1	(reference)		49	11	1	(reference)	
positive	33	90	0.927	(0.466–1.847)	0.83	95	28	0.762	(0.350–1.658)	0.492
Family Tumor History	50	133				144	39			
no	8	114	1	(reference)		121	35	1	(reference)	
yes	42	19	1.143	(0.465–2.807)	0.771	23	4	1.663	(0.539–5.031)	0.372
Procreative Times	47	128				138	37			
< 1	37	105	1	(reference)		112	30	1	(reference)	
≥ 1	10	23	0.81	(0.353–1.862)	0.62	26	7	1.005	(0.398–2.539)	0.991
Progestrone Receptor	50	133				144	39			
negative	22	53	1	(reference)		59	16	1	(reference)	
positive	28	80	0.843	(0.437–1.627)	0.611	85	23	1.002	(0.488–2.058)	0.995
Tumor Location	50	133				144	39			
left	22	62	1	(reference)		66	18	1	(reference)	
right	28	69	1	(reference)		77	20	1	(reference)	
both	0	2	---	---	0.631	1	1	---	---	0.603
Tumor Size (cm)	50	133				144	39			
≤ 3	24	70	1	(reference)		76	18	1	(reference)	
> 3	26	63	1.204	(0.628–2.308)	0.576	68	21	0.767	(0.377–1.559)	0.463
Tumor Type	50	133				144	39			
Infiltrating ductal carcinoma	47	118	1	(reference)		128	37	1	(reference)	
others	3	15	1.992	(0.551–7.198)	0.285	16	2	0.432	(0.095–1.967)	0.266
Incipience/Recurrence	49	133				144	38			
Incipience	22	87	1	(reference)		92	17	1	(reference)	
Recurrence	27	46	2.321	(1.192–4.521)	0.012*	52	21	0.458	(0.222–0.944)	0.032*
Lymph node metastasis	49	131				141	39			
no	29	76	1	(reference)		83	17	1	(reference)	
yes	20	55	0.953	(0.489–1.857)	0.887	58	22	0.904	(0.442–1.851)	0.783
Menopause	50	133				144	39			
no	27	88	1	(reference)		87	28	1	(reference)	
yes	23	45	1.666	(0.859–3.230)	0.129	57	11	1.668	(0.770–3.614)	0.192
Primiparous Age	47	129				139	37			
< 30	45	125	1	(reference)		136	34	1	(reference)	
≥ 30	2	4	0.72	(0.127–4.006)	0.709	3	3	4	(0.773–20.70)	0.076

**Table 6B:** The Associations between *TSPYL6* polymorphisms and clinical characteristics of breast cancer patients

Variables	rs11896604					rs843706				
	GG + CG	CC	OR^a^	95% CI	*P*^b^	CA + CC	AA	OR^a^	95% CI	*P*^b^
Age	55	128				144	39			
≤ 40	16	41	1	(reference)		41	16	1	(reference)	
> 40	39	87	1.149	(0.576–2.291)	0.694	103	23	1.748	(0.839–3.639)	0.133
Age of Menarche	55	128				144	39			
≤ 12	6	19	1	(reference)		22	3	1	(reference)	
> 12	49	109	0.702	(0.264–1.868)	0.477	122	36	2.164	(0.612–7.646)	0.221
BMI	55	128				144	39			
≤ 24	38	83	1	(reference)		98	23	1	(reference)	
> 24	17	45	0.825	(0.419–1.624)	0.578	46	16	0.675	90.326–1.397	0.288
Breastfeeding Duration	51	119				133	37			
≤ 6	3	9	1	(reference)		10	2	1	(reference)	
> 6	48	110	0.764	(0.198–2.946)	0.695	123	35	1.423	(0.298–6.797)	0.657
Clinical Stages	55	128				144	39			
I/II	43	92	1	(reference)		112	23	1	(reference)	
III/IV	12	36	0.713	(0.338–1.505)	0.374	32	16	0.411	(0.194–0.869)	0.018**
Estrogen Receptor	55	128				144	39			
negative	19	41	1	(reference)		49	11	1	(reference)	
positive	36	87	0.893	(0.458–1.742)	0.74	95	28	0.762	(0.350–1.658)	0.492
Family Tumor History	55	128				144	39			
no	45	111	1	(reference)		121	35	1	(reference)	
yes	10	17	1.151	(0.617–3.410)	0.391	23	4	1.663	(0.539–5.131)	0.372
Procreative Times	52	123				138	37			
< 1	41	101	1	(reference)		112	30	1	(reference)	
≥ 1	11	22	0.812	(0.361–1.824)	0.614	26	7	1.005	(0.398–2.539)	0.991
Progestrone Receptor	55	128				144	39			
negative	25	50	1	(reference)		59	16	1	(reference)	
positive	30	78	0.769	(0.406–1.457)	0.42	85	23	1.002	(0.488–2.058)	0.995
Tumor Location	55	128				144	39			
left	25	59	1	(reference)		66	18	1	(reference)	
right	30	67	1	(reference)		77	20	1	(reference)	
both	0	2	---	---	0.638	1	1	---	---	0.603
Tumor Size (cm)	55	128				144	39			
≤ 3	27	67	1	(reference)		76	18	1	(reference)	
> 3	28	61	1.139	(0.605–2.144)	0.686	68	21	0.767	(0.377–1.559)	0.463
Tumor Type	55	128				144	39			
Infiltrating ductal carcinoma	52	113	1	(reference)		128	37			
others	3	15	2.301	(0.638–8.295)	0.192	16	2	0.432	(0.095–1.967)	0.266
Incipience/Recurrence	54	128				144	38			
Incipience	24	85	1	(reference)		92	17	1	(reference)	
Recurrence	30	43	2.471	(1.290–4.734)	0.006*	52	21	0.458	(0.222–0.944)	0.032*
Lymph node metastasis	54	126				141	39			
no	33	72	1	(reference)		83	22	1	(reference)	
yes	21	54	0.848	(0.442–1.627)	0.621	58	17	0.904	(0.442–1.851)	0.783
Menopause	55	128				144	39			
no	32	83	1	(reference)		87	28	1	(reference)	
yes	23	45	1.326	(0.694–2.532)	0.393	57	11	1.668	(0.770–3.614)	0.192
Primiparous Age	52	124				139	37			
< 30	50	120	1	(reference)		136	34	1	(reference)	
≥ 30	2	4	0.833	(0.148–4.697)	0.836	3	3	4	(0.773–20.70)	0.076

BMI: body mass index; CI: confidence interval. OR: odds ratio.

a Adjusted for Age, Age of Menarche, BMI, Breastfeeding Duration, Clinical Stages, Estrogen Receptor, Family Tumor History, Procreative Times, Progestrone Receptor, Tumor Location, Tumor Size (cm), Tumor Type, Incipient/Recurrence, Lymph node metastasis, Menopause and Primiparous Age.

b Two-sided Chi-square test for the distributions of genotype frequencies.

* *p* < 0.05 indicates statistical significance.

## DISCUSSION

In this study, we investigated the association between SNPs in the TSPYL6 gene and BC risk in Han Chinese women. We found that four SNPs (rs11896604, rs843706, rs11125529, and rs843711) were associated with the risk of BC in this population. Rs11896604 was associated with a decreased risk of BC in a dominant and genotype model, but the various genotypes were associated with an increased risk of recurrence in BC patients. An association between this locus and other diseases has not been previously reported. Rs843706 was associated with an increased risk of BC in a recessive model, but there was a decreased association between the SNP and the risk of recurrence as well as with clinical stage III/IV BC.

We are the first to demonstrate an association between this locus and BC susceptibility. Rs11125529 was associated with a decreased risk of BC in a genotype model, but an increased risk of recurrence. Although Ding et al. reported neither the genotype nor the allele frequencies at rs11125529 in *ACYP2* differed significantly between coronary heart disease patients and normal controls [13]. The association between the telomere length-related variant rs11125529 in *ACYP2* and gastric cancer risk was previously investigated in a Chinese population, but no significant association was identified [14]. We found that the rs843711 genotypes in the *TSPYL6* gene were inversely correlated with clinical stage III/IV BC. Finally, rs843645 and rs12615793 were not associated with the risk of BC.

The function of *TSPYL6* may be similar to those of other members of the TTSN superfamily. However, the molecular mechanisms underlying *TSPYL6* function have not been elucidated. Mutation of *TSPYL* can cause sudden infant death with dysgenesis of the testes (SIDDT) in affected males, indicating that *TSPYL* is important for the development of the testis and other tissues such as the brain [15]. Although *TSPYL* is expressed in all tissues [16], the role of *TSPYL* in tumor cells is not clear. The *TSPYL4* gene is located 25 kb from *TSPYL*, however no coding variants were identified in affected individuals with direct sequencing. The *TSPYL1* gene does not contain any introns, but the exact composition has not been determined [17].

*TSPYL2* gene and cyclin B can inhibit cell proliferation by arresting cell growth in response to DNA damage [18]. Thus, it has been suggested that *TSPYL2* is a negative regulator of cell cycle progression. The *TSPYL2* gene is silenced in glioma and malignant lung tissue, and in certain lung cancer cell lines [19]. Overexpression of *TSPYL2* can inhibit human lung and breast cancer cell lines [20]. However, there is limited evidence for a direct function of *TSPYL2* in cell cycle control. Interestingly, the *TSPYL5* gene has been reported to suppress gastric cancer development [21]. Further studies are required to characterize the function of *TSPYL6* and elucidate the mechanisms underlying the association between the *TSPYL6* and BC susceptibility. Currently, the relationship between clinical characteristics in BC patients and *TSPYL6* gene expression/function is not clear.

Our study is the first to demonstrate that polymorphisms in *TSPYL6* affect the pathogenesis of BC and are associated with clinicopathological characteristics of BC patients. Collectively, the results provide insight into the pathogenesis of BC. Although this study had sufficient statistical power, there were still some intrinsic limitations. First, the sample size was relatively small (183 cases and 195 controls). Therefore, our findings must be confirmed in studies with larger sample sizes as well as in a meta-analysis. Additionally, we only analyzed Han Chinese women. Therefore, our results must be validated in studies of other populations. Finally, although we identified significant associations between four SNPs (rs11896604, rs843706, rs11125529, and rs843711) and BC susceptibility, the mechanisms responsible for the associations are still unclear. Further studies of *TSPYL6* and other members of the TTSN superfamily are necessary to dissect the mechanisms by which polymorphisms in these genes contribute to BC risk. Hereditary, endocrine, environmental, and life style factors should be also considered.

We performed Bonferroni correction in our statistical analysis, but found no statistical significant associations between *TSPYL6* SNPs and risk of BC. This may be due to the relatively small sample size, the selection criteria for *TSPYL6* SNPs (minor allele frequency [MAF] > 5%), and the weakness of Bonferroni correction itself (the interpretation of a finding depends on the number of other tests performed). True differences may have been deemed non-significant given the likelihood of type II errors.

## MATERIALS AND METHODS

### Study participants

A total of 183 patients with BC and 195 healthy women were included in this study. The patients were treated at the Second Affiliated Hospital of Xi'an Jiao Tong University between January 2013 and November 2015. All demographic and related clinical data including residential region, age, ethnicity, and education status were collected through a face-to-face questionnaire and a review of medical records. The clinical and demographic characteristics of the patients are shown in Table 1. Patients who had been recently diagnosed with primary BC (confirmed by histopathological analysis) were included in the study. Patients diagnosed with other types of cancers or who underwent radiotherapy or chemotherapy were excluded. Control patients who had undergone annual health evaluations were recruited from health checkup centers affiliated with our institution. All controls were matched with cases based on age (*p* = 0.218) and ethnicity. All control patients had no history of cancer. Factors that could influence the mutation rate were minimized. The participants were women who were ≥ 18 years old with good mental health and no blood relatives with BC going back three generations. This study was performed in accordance with the Chinese Department of Health and Human Services regulations for the protection of human research subjects. Informed consent was obtained from all participants and the study protocols were approved by the Institutional Review Board of Xi'an Jiao Tong University.

### SNP selection and genotyping

Validated SNPs that had a MAF > 5% in the HapMap Asian population were selected for the association analysis [12, 20, 22, 23]. Venous blood samples (5 mL) were collected from each patient during a laboratory examination. DNA was extracted from whole blood samples using the Gold Mag-Mini Whole Blood Genomic DNA Purification Kit (version 3.0; TaKaRa, Japan) [24]. The DNA concentration was measured by spectrometry (DU530 UV/VIS spectrophotometer, Beckman Instruments, Fullerton, CA, USA). The Sequenom MassARRAY Assay Design 3.0 software (Sequenom, Inc, San Diego, CA, USA) was used to design the multiplexed SNP Mass EXTEND assay. Genotyping was performed using a Sequenom MassARRAY RS1000 (Sequenom, Inc.) according to the manufacturer's protocol [25]. The SequenomTyper 4.0 Software™ (Sequenom, Inc.) was used to manage and analyze the data [26]. The primers corresponding to each SNP are shown in Table 7. Based on these results, the following six SNPs were selected: rs843645, rs11125529, rs12615793, rs843711, rs11896604, and rs843706. The SNP data are shown in Table 3.

**Table 7:** Primers used for this study

SNP_ID	1st-PCRP	2nd-PCRP	UEP_SEQ
rs843645	ACGTTGGATGGAAATCTGA ATACCACCTAC	ACGTTGGATGACAGTGCCTTTA GCAAGGTG	TCATAGGCACTACT GTATC
rs11125529	ACGTTGGATGGAGCTTAGTT GTTTACAGATG	ACGTTGGATGCCGAAGAAAAG AAGATGAC	AGAAAAGAAGATG ACTAAAACAT
rs12615793	ACGTTGGATGTTTGAGCTTAG TTGTTTAC	ACGTTGGATGATCTTGGCCCTT GAAGAA	AAATTGAGTGACAA| ATATAAACTAC
rs843711	ACGTTGGATGGACAAAGGACC TTACAACTC	ACGTTGGATGTGCCTTGTGGGA ATTAGAGC	gggaTCAGGGAACCA GTGCAAA
rs11896604	ACGTTGGATGAAGTCAGAATA GTGCTTAC	ACGTTGGATGTGTCTCTGACCT AGCATGTA	GTTAAGCTTGCAA GGAG
rs843706	ACGTTGGATGTGAAAGCCAT AAATATTTTG	ACGTTGGATGTGAATAACTTGG TCTTATC	cACTTGGTCTTATCT GATGC

### Statistical analysis

Chi-squared tests (categorical variables) and Student's *t-*tests (continuous variables) were used to evaluate the differences in the demographic characteristics between the cases and controls [27]. The Hardy-Weinberg equilibrium of each SNP was assessed in order to compare the expected frequencies of the genotypes in the control patients. All of the minor alleles were regarded as risk alleles for BC susceptibility. To evaluate associations between the SNPs and risk of BC in the four models (genotype, dominant, recessive, and additive), ORs and 95% CIs were calculated using unconditional logistic regression analysis [28]. In multivariate analyses, unconditional logistic regression was used to assess the association between each SNP and the risk of BC after adjusting for BMI, age, and menopause [28]. Linkage disequilibrium analysis and SNP haplotypes were analyzed using the Haploview software package (version 4.2) and the SHEsi software platform (http://www.nhgg.org/analysis/) [29]. All statistical analyses were performed using the SPSS version 17.0 statistical package (SPSS, Chicago, IL, USA) and Microsoft Excel. A *p* < 0.05 was considered statistically significant and all statistical tests were two-sided.

## CONCLUSIONS

In summary, we have identified four novel associations between SNPs (rs11896604, rs843706, rs11125529, and rs843711) in *TSPYL6* and BC. Our results suggest that these SNPs may contribute to BC development and possibly other complex genetic traits. These SNPs may function as molecular markers of BC susceptibility, and could therefore be used as diagnostic and prognostic markers in clinical studies of BC patients.
